# Heavy metals immobilization and improvement in maize (*Zea mays* L.) growth amended with biochar and compost

**DOI:** 10.1038/s41598-021-97525-8

**Published:** 2021-09-16

**Authors:** Muhammad Irfan, Muhammad Mudassir, Muhammad Jamal Khan, Khadim Muhammad Dawar, Dost Muhammad, Ishaq Ahmad Mian, Waqas Ali, Shah Fahad, Shah Saud, Zafar Hayat, Taufiq Nawaz, Shah Alam Khan, Sartaj Alam, Beenish Ali, Jan Banout, Sagher Ahmed, Sidra Mubeen, Subhan Danish, Rahul Datta, Abdallah M. Elgorban, Raf Dewil

**Affiliations:** 1 Department of Soil and Environmental Sciences, The University of Agriculture, Peshawar, Pakistan; 2 Hainan Key Laboratory for Sustainable Utilization of Tropical Bioresource, College of Tropical Crops, Hainan University, Haikou, 570228 Hainan China; 3 Department of Agronomy, The University of Haripur, Haripur, 22620 Pakistan; 4 Department of Horticulture, Northeast Agriculture University, Harbin, China; 5 Department of Agriculture, Abdul Wali Khan University Mardan, Khyber Pakhtunkhwa, 23200 Pakistan; 6 Department of Plant Protection, The University of Agriculture Peshawar, Khyber Pakhtunkhwa, 25120 Pakistan; 7 Department of Food Science and Technology, The University of Agriculture Peshawar, Khyber Pakhtunkhwa, 25120 Pakistan; 8 Department of Geology, Bacha Khan University, Charsadda, Pakistan; 9 Faculty of Trophical AgriSciences, Czech University of Life Sciences, Prague, Czechia; 10 Department of Soil Science, Faculty of Agricultural Sciences and Technology, Bahauddin Zakariya University, Multan, 60800 Pakistan; 11 Department of Agronomy, Faculty of Agricultural Sciences and Technology, Bahauddin Zakariya University, Multan, 60800 Pakistan; 12 Department of Chemistry, The Women University Multan, Punjab, 60000 Pakistan; 13 Department of Geology and Pedology, Faculty of Forestry and Wood Technology, Mendel University in Brno, Zemedelska1, 61300 Brno, Czech Republic; 14 Department of Botany and Microbiology, College of Science, King Saud University, Riyadh, 11451 Saudi Arabia; 15 Department of Chemical Engineering, Process and Environmental Technology Lab, KU Leuven (University of Leuven), Leuven, Belgium

**Keywords:** Plant stress responses, Abiotic, Plant sciences, Environmental sciences, Pollution remediation

## Abstract

Soil with heavy metals contamination, mainly lead (Pb), cadmium (Cd), and chromium (Cr) is a progressively worldwide alarming environmental problem. Recently, biochar has been used as a soil amendment to remediate contaminated soils, but little work has been done to compare with other organic amendments like compost. We investigated biochar and compost's comparative effect on Pb, Cd, and Cr immobilization in soil, photosynthesis, and growth of maize plants. Ten kg soil was placed in pots and were spiked with Pb, Cd, and Cr at concentrations 20, 10, 20 mg kg^−1^. The biochar and compost treatments included 0, 0.5, 1, 2, and 4% were separately applied to the soil. The crop from pots was harvested after 60 days. The results show that the highest reduction of AB-DTPA extractable Pb, Cd, and Cr in soil was 79%, 61% and 78% with 4% biochar, followed by 61%, 43% and 60% with 4% compost compared to the control, respectively. Similarly, the highest reduction in shoot Pb, Cd, and Cr concentration was 71%, 63% and 78%with 4% biochar, followed by 50%, 50% and 71% with 4% compost than the control, respectively. The maximum increase in shoot and dry root weight, total chlorophyll contents, and gas exchange characteristics were recorded with 4% biochar, followed by 4% compost than the control. The maximum increase in soil organic matter and total nitrogen (N) was recorded at 4% biochar application while available phosphorus and potassium in the soil at 4% compost application. It is concluded that both biochar and compost decreased heavy metals availability in the soil, reducing toxicity in the plant. However, biochar was most effective in reducing heavy metals content in soil and plant compared to compost. In the future, more low-cost, eco-friendly soil remediation methods should be developed for better soil health and plant productivity.

## Introduction

Soil contamination has been recognized progressively as a global environmental problem^1–5^. Worldwide, fast population growth, technology progresses, and mechanization is the anthropogenic happenings such as mining, smelting, and industrial operations, dumping of different unwanted raw materials, excessive use of inorganic fertilizer as well as the use of pesticides are the major attributes to soil heavy metals contamination^6–10^. Also, improper ways by which different waste includes hospitals, agricultural, industrials, and household waste, are disposed of severely contaminate the soil. Industrial effluent by which crop is irrigated due to shortage of freshwater for irrigation in developing countries like Pakistan may be a potential risk for humans by contaminating soil^11–16^. These wastewaters have a substantial concentration of toxic metals. Accumulation of excess heavy metals in soil adversely affects soil properties and plant growth^3–5,13,14,17,18^. Thus, make the soil unsafe for crop production^4,13,15,18–20^.

The nature of toxic metals, as they are non-biodegradable, remain in the soil environment for a long time and accumulates in living bodies, causing numerous disorders in the body. Heavy metals, particularly lead (Pb = 250–500 ppm), cadmium (Cd = 3–6 ppm), and chromium (Cr ≥ 150 ppm) high concentration in the soil environment^21–23^, were considered as a major threat to human health through the food chain cause severe harm to the environment^24,25^. A threshold level of chromium in water is 0.05 mg per liter in water, whereas any lead level can have a toxic effect on humans. Harvesting of the agricultural crop on polluted soils causes a reduction in soil productivity and affects plant yield^26^. The accumulation of heavy metals causes disorders in plant growth developments, including plant respiration, photosynthetic rate, and transpiration, resulting in reduced plant growth and crop production^26–30^.

Worldwide, especially in Pakistan, agricultural land is decreasing due to several reasons, including coal and chromite mining activities and wastewater irrigation, requiring special attention. Thus, exploring environmentally and economically friendly methods reduces heavy metal’s bioavailability and ensures food security is an urgent need. For these purposes, various scientists have suggested multiple organic and inorganic strategies to achieve that goal^31,32^. These organic and inorganic methods included compost material, farmyard manure, poultry manure, biochar and gypsum, elemental sulfur and Diammonium Phosphate (DAP) etc. Organic amendments incorporation in contaminated soil benefits over inorganic due to cost-effectiveness, higher biodegradability, and enhancement in soil properties^33^. It has been reported that the accumulation of toxic metals was significantly decreased, and maize growth, biomass production increased with biochar application^34^. Biochar and compost combination has also been reported as potential soil amendments for reducing bioavailable fractions of Pb, Zn, and Cd in the soil^35^.

Biochar is a substance rich in carbon and can be derived from various feedstocks under thermal conditions in a limited supply of oxygen called pyrolysis technology^36^. Biochar became more carbonaceous as the temperature was increased, and the carbon content range from 58 to 64% at the temperature range of 300 to 700 °C^36^. Biochar interacts with heavy metals through different mechanisms like surface adsorption, exchange, surface complexation, and precipitation characteristics^28,37,38^. The potential of biochar to immobilize the heavy metals in soil depends on its acid neutralization ability and high cation exchange capacity^39^. Biochar has unique characteristics of high surface area and high adsorption capability, making them more prone to hold inorganic pollutants^37^. By direct and indirect effect on contaminated soil properties, biochar may increase soil's potential to produce high crop yield and improve soil characteristics^34^.

Compost is a stable humic substance it helps in accelerating soil microbial and enzyme activities. It is a commonly used soil amendment in poor urban areas due to its low cost and convenience^40,41^. Compost is known to increase soil organic matter and improve soil structure; thus, there is enhanced plant growth. In addition, soil amendments such as biochar compost and their mixture can also immobilize heavy metals. Although there are several studies on the role of biochar and compost in immobilizing heavy metal, Still, little work has been done mainly on Pakistani soil to compare the sole effect of both organic amendments (biochar and compost) on the immobilization of heavy metals like Pb, Cd, and Cr in soil and their toxicity to plants. The objective of this study was to investigate biochar and compost's comparative effect on Pb, Cd, and Cr immobilization in soil, photosynthesis, and maize growth. It is hypothesized that biochar and compost can decrease the toxicity of heavy metals in maize by remediating the soil contamination. Our study will potentially help to convey scientific knowledge of biochar uses and their potential benefits to policymakers and practitioners. The study will directly help farmers, gardeners in increasing plant productivity and saving money.

## Results and discussion

### Effect of biochar and compost on Pb, Cd and Cr immobilization

Biochar and compost lonely added to soil at the levels of 0.5, 1, 2 and 4% to artificially contaminated soil significantly (p < 0.05) reduced the availability of Pb, Cd and Cr, respectively (Table 1). The Pb content in soil (control) was decreased from 18.26 to 3.82 mg kg^−1^ at 4% biochar application, followed by 4% compost. Cd concentration in soil was decreased from 9.33 mg kg^−1^ (control) to 2.67 mg kg^−1^ at 4% biochar application, followed by 2% biochar and 4% compost. The Cr content was decreased at 4% biochar application from 18.47 mg kg^−1^ (control) to 4.04 mg kg^−1^ followed by 4% compost and 2% biochar.

**Table 1:** Biochar and compost impact on Pb, Cd and Cr concentration in soil.

Treatments	Pb (mg kg^−1^)	Cd (mg kg^−1^)	Cr (mg kg^−1^)
Control	18.26 a	9.33 a	18.47 a
BC0.5%	15.94 c	8.07 c	16.16 c
BC1%	12.04 e	6.44 f.	12.26 e
BC2%	7.58 g	4.50 h	7.81 g
BC4%	3.82 i	2.67 i	4.04 i
CP0.5%	16.67 b	8.67 b	16.88 b
CP1%	14.25 d	8.06 d	14.47 d
CP2%	10.71 f	6.87 e	10.93 f
CP4%	7.13 h	5.36 g	7.34 h

*BC* Biochar, *CP* Compost.

.

The statistical results indicated that biochar and compost consistently reduced soil Pb, Cd, and Cr concentrations at different rates. The reduction amount of Pb, Cd, and Cr in soil by each biochar level was greater than the compost level. This means that biochar is more efficient in stabilizing the Pb, Cd, and Cr in soil than compost. Different postulated mechanisms like surface adsorption, precipitation, electrostatic interaction, and ion exchange through which biochar particles interact with heavy metals and cause immobilization. Therefore, biochar’s unique characteristics, like high surface area and more specific groups and alkaline pH, play an important role in heavy metals sorption. Our results indicated that biochar application increased soil pH and the prevalence of electrostatic interaction in heavy metals adsorption depends on solution pH. Biochar addition stabilized heavy metals content due to its major characteristics like surface heterogeneity, different functional groups, and a large surface area that adsorbed the heavy metals on the soil surface. Another possible biochar mechanism to adsorb heavy metals on the micro-porous structure and excess soluble salts enhanced the heavy metal’s immobilization by precipitation and surface sorption. The application of biochar potentially adsorbed heavy metals by bonding with oxygenated functional groups^42^. A more recent study demonstrated that organic amendments like biochar, compost, and farmyard manure decreased Cd availability in soil and decreased concentration in different parts of wheat such as shoots, roots, husks, and grains^43^. Applying biochar significantly reduced AB-DTPA-extractable heavy metal concentrations of soils, indicating metal immobilization^34^.

### Effect of biochar and compost on Pb Cd and Cr content in maize plants

Both organic amendments (biochar and compost) significantly (p < 0.05) decreased the Pb, Cd, and Cr concentrations in maize shoots (Table 2). The incorporation of biochar and compost progressively decreased Pb, Cd, and Cr in the soil; consequently, the amounts of Pb, Cd, and Cr in maize plants were decreased. The concentration of Pb, Cd and Cr in shoot were decreased from 2.58, 1.95 and 2.95 mg kg^−1^ (control) to 0.76, 0.72 and 0.64 mg kg^−1^ at 4% biochar. The data shows that the highest reduction of Pb, Cd, and Cr in maize plants was recorded at the highest biochar application rate. Also, incremental biochar and compost rates consistently reduced Pb, Cd, and Cr concentration in maize plants. The biochar effect was more pronounced in reducing Pb, Cd, and Cr concentration in maize plants than compost. The same results were also reported^34,43^. The addition of biochar strongly phyto-stabilize the heavy metals content in maize plant shoots and enhanced the quality of contaminated soil by reducing phytotoxicity. The results are in line with^44^ exhibited that biochar's application reduced heavy metal’s uptake and improved the fixed fraction of heavy metals.

**Table 2:** Biochar and compost impact on Pb, Cd, and Cr concentration in maize shoot.

Treatments	Pb (mg kg^−1^)	Cd (mg kg^−1^)	Cr (mg kg^−1^)
Control	2.58 a	1.95 a	2.95 a
BC0.5%	2.32 c	1.78 c	2.18 b
BC1%	1.84 e	1.49 e	1.55 e
BC2%	1.27 i	1.13 g	1.08 g
BC4%	0.76 j	0.72 i	0.64 i
CP0.5%	2.41 b	1.83 b	2.01 c
CP1%	2.11 d	1.61 d	1.67 d
CP2%	1.71 g	1.31 f	1.27 f
CP4%	1.28 h	0.98 h	0.86 h

*BC* Biochar, *CP* Compost.

### Impact of biochar and compost on soil chemicals properties

The pH of soil increased significantly (p < 0.05) with biochar and compost incorporation into the soil (Table 3). Soil pH increased from 7.53 (control) to 7.96 at 4% biochar application, followed by 4% compost and 2% biochar. There was a slight increase in soil pH with increasing levels of both biochar and compost. Generally, biochar produced at high temperature attend the alkaline pH, and the alkaline nature of biochar may be due to the separation of alkali salts during pyrolysis of biomass^36^. According to Al-Wabel et al.^34^, applying biochar at a 5% rate resulted in a 0.16–0.17 unit increase in soil pH.

**Table 3:** Biochar and compost impact on soil chemical properties.

Treatments	pH –	EC (dSm^−1^)	Organic matter (%)	N (%)	P (mg kg^−1^)	K (mg kg^−1^)
Control	7.53 ± 0.006 h	0.14 ± 0.014 f	1.09 ± 0.010 i	0.22 ± 0.010 i	1.12 ± 0.80 i	38.29 ± 1.62 i
BC0.5%	7.73 ± 0.007 e	0.17 ± 0.006 d	1.22 ± 0.008 g	0.27 ± 0.006 h	1.49 ± 0.65 h	51.84 ± 2.42 h
BC1%	7.81 ± 0.012 e	0.18 ± 0.012 c	1.30 ± 0.012 e	0.32 ± 0.014 f	1.78 ± 0.42 f	61.44 ± 3.20 f
BC2%	7.83 ± 0.006 b	0.19 ± 0.008 b	1.46 ± 0.014 c	0.38 ± 0.008 d	2.11 ± 0.60 e	89.28 ± 2.64 d
BC4%	7.96 ± 0.012 a	0.21 ± 0.012 a	1.72 ± 0.012 a	0.45 ± 0.010 a	2.29 ± 0.82 d	103.45 ± 1.28 b
CP0.5%	7.65 ± 0.018 f	0.15 ± 0.014 e	1.21 ± 0.014 h	0.28 ± 0.012 g	1.51 ± 0.46 g	54.21 ± 3.26 g
CP1%	7.72 ± 0.008 e	0.17 ± 0.008 d	1.27 ± 0.006 f.	0.35 ± 0.008 e	2.84 ± 0.68 c	67.37 ± 1.60 e
CP2%	7.79 ± 0.012 d	0.18 ± 0.010 c	1.34 ± 0.014 d	0.41 ± 0.012 c	3.19 ± 0.55 b	96.53 ± 2.26 c
CP4%	7.84 ± 0.006 b	0.20 ± 0.006 b	1.52 ± 0.008 b	0.43 ± 0.010 b	5.11 ± 0.64 a	109.34 ± 3.50 a

*BC* Biochar, *CP* Compost.

Soil EC was increased to 0.21 dS m^−1^ from 0.14 dS m^−1^ (control) at 4% biochar followed by 4% compost and 2% biochar (Table 3). It was reported by^34^ that soil treated with biochar, compost, and farmyard manure significantly increased soil EC compared to untreated soil. Our results show that initial EC values of experimental soil, biochar, and compost are very low, which may not create soil problems. It is also very important to know the EC value of biochar before applying it to cropland to avoid creating a soil salt problem, which would adversely affect plant growth^36^.

The results showed that organic matter was significantly (p < 0.05) increased by both biochar and compost levels (Table 3). The lowest organic matter (1.09%) was noted in control, while the highest organic matter (1.72%) was noted at 4% biochar application, followed by 4% compost. Consistently, organic matter was increased with increasing levels of both biochar and compost. Both biochar and compost are rich sources of C, which can restore soil deficient in organic matter. Naeem et al.^45^ reported that biochar prepared at 500 °C pyrolysis temperature increased the carbon content of typic calciargid soil in Pakistan.

The results revealed that compared to biochar, compost application significantly (p < 0.05) increased total N and available P and K content in soil (Table 3). Also, different levels of biochar consistently increased total N and available P and K content. The concentration of N, P, and K in the biochar seems to be low compared to compost; therefore, compost has a more significant effect on total N and available P and K content. Generally, biochar has low N content, which could be due to an increase in temperature that causes the volatilization of N during pyrolysis processes.

### Effect of biochar and compost on chlorophyll contents, gas exchange characteristics and maize growth

**Figure 1:**
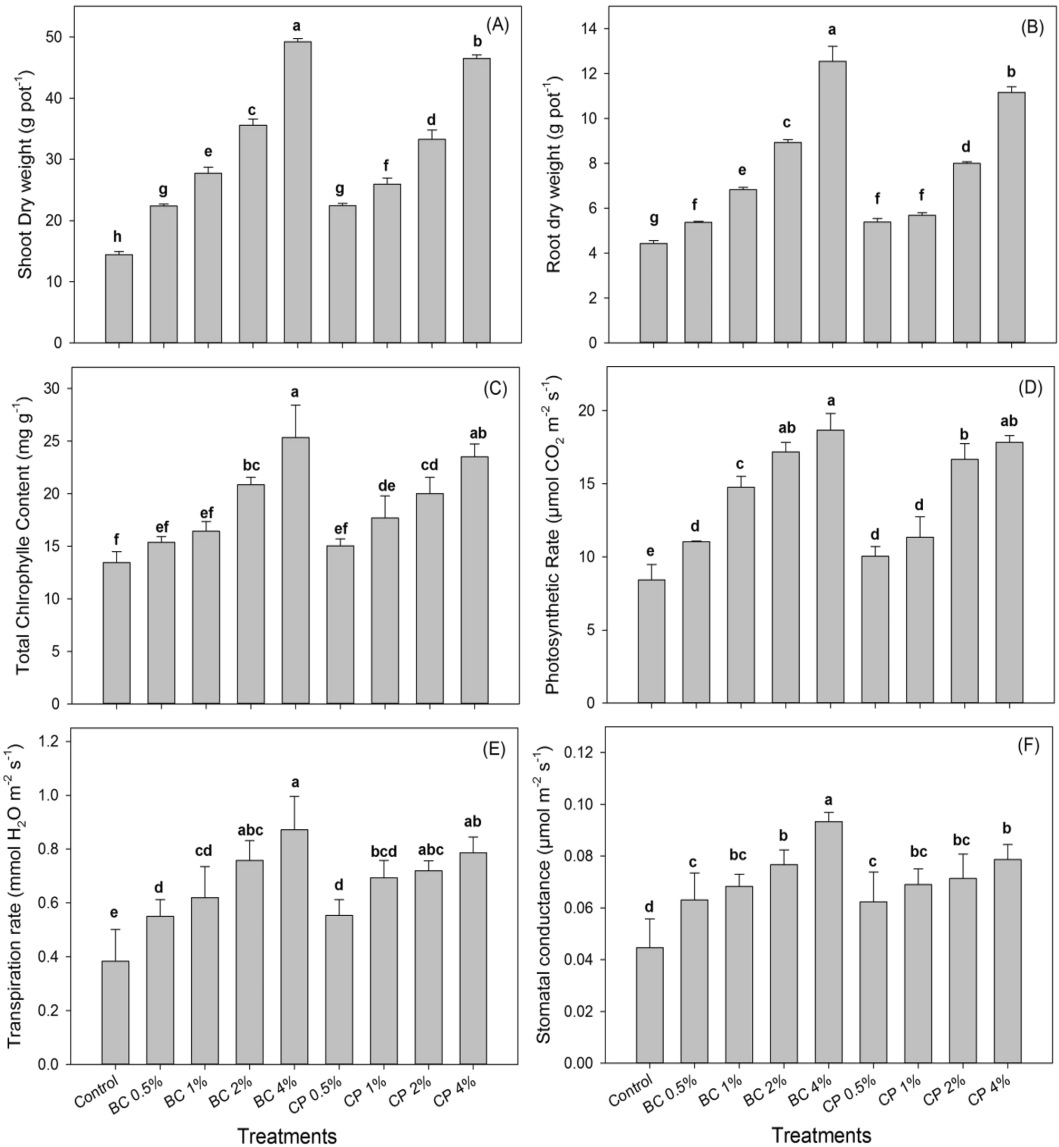
Effect of biochar and compost on chlorophyll contents, gas exchange characteristics, and maize growth.

Data regarding chlorophyll contents, gas exchange characteristics, and maize growth are presented (Fig. 1). Total chlorophyll contents, photosynthetic rate, transpiration rate, and stomatal conductance (p < 0.05) increased with biochar and compost application compared with control. The lowest values of total chlorophyll contents, photosynthetic rate, transpiration rate, and stomatal conductance were recorded in control plants. The highest values were recorded in plants amended with 4% biochar fallowed by the compost's highest application rate. The increase in total chlorophyll contents was (92%), photosynthetic rate (138%), transpiration rate (125%), and stomatal conductance (125%) with an application of 4% biochar when compare with control.

Furthermore, it was noted that increasing rates of both organic amendments (biochar and compost) increased the chlorophyll contents and gas exchange characteristics. Similarly, dry shoot and root weights were significantly (p < 0.05) improved by applying biochar and compost under Pb, Cd, and Cr stress. Application of biochar at a 4% rate increased dry shoot and root weight by 250% and 225%, respectively, compared to control. A severe plant growth reduction was noted in soil spiked with Pb, Cd, and Cr without biochar and compost application.

Overall, significantly more chlorophyll contents, gas exchange characteristics, and maize growth by each biochar level were noted compared to compost levels. The improvement in maize plant’s growth parameters might be due to soil fertility improvements like N and organic matter improved and/or phytostabilization of Pb, Cd, and Cr with these organic amendments. The reduction in chlorophyll content under metal stress might be due to the distortion of the chloroplast. It has been reported that organic amendments, including biochar and compost, increased chlorophyll contents and gas exchange characteristics compared to untreated soil (control)^46^. Biochar may indirectly improve plant growth by improving soil physical properties such as water-holding capacity and soil structure. Plant growth on contaminated soil can be enhanced by such organic amendment and to reduce the toxicity of elements such as Pb, Cd, and Cr in the plant. The previous finding showed that plant growth under metal stresses increased with organic amendments^47,48^. Improvement of K status is required to reduce Cd toxicity in rice seedlings^49^. Furthermore, the application of essential nutrients such N, P, K, Mg, Zn, and Fe in Cd contaminated soils has recommended counteracting Cd toxicity in the plant.

## Conclusions

It is concluded that biochar and compost organic amendments can stabilize Pb, Cd, and Cr in soil and reduce its toxicity in maize plants. But biochar was most effective by immobilizing Pb, Cd, and Cr in the soil compared to compost. Furthermore, in terms of plant growth, soil organic matter, and N content in the soil, biochar amendments showed superiority over the compost. Long-term experiments need to be conducted to assess these amendment’s stability for situ immobilization of Pb, Cd, and Cr in soil. Biochar should be efficiently used to remediation heavy metal pollution in the soil and improve plant productivity. In the future, more low-cost, eco-friendly soil remediation methods should be developed for better soil health and plant productivity.

## Materials and methods

### Soil sampling

At the beginning of the pot experiment, a bulk soil sample (0–20 cm) was taken from the research farm, The University of Agriculture Peshawar. The bulk materials such as crop residues and roots were removed from bulk soil. Then soil was grounded with a hammer and passed through a 2 mm sieve after air drying. The soil used in the pot experiment was examined earlier before starting the experiment for basic physical and chemical properties (Table 4).

**Table 4:** Physical and chemical properties of soil used in the experiment.

Physical/chemical properties	Values	References
Sand	35%	^50^
Silt	40%	
Clay	25%	
Texture class	Loam	
pH	7.5	^51^
Electrical conductivity (EC)	0.15 (dSm^−1^)	^52^
Soil organic matter	1.12%	^53^
Total N	0.21%	^54^
P	1.14 mg kg^−1^	^55^
K	43.4 mg kg^−1^	^56^
Pb	2.72 mg kg^−1^	^57^
Cd	0.10 mg kg^−1^	
Cr	0.32 mg kg^−1^	

### Biochar and compost characteristics

Maize straw-derived biochar and prepared compost in the Department of Soil and Environmental Sciences, The University of Agriculture Peshawar, were used in the experiment. Biochar was prepared in a muffle furnace at 550 °C under a limited supply of oxygen. Before use in the experiment, biochar and compost were grounded and passed through 2 mm sieve. The pH and EC were determined in 1:10 the ratio of biochar/compost to water suspension. The pH of biochar and compost (7.30 and 7.19), EC (0.15 and 0.14 dSm^−1^), and organic C (40 and 24%), respectively. Heavy metals content in biochar and compost were not detected. Biochar (Cd = 0.29 mg kg^−1^, Pb = 3.20 mg kg^−1^ and Cr = 0.26 mg kg^−1^) and compost (Cd = 0.09 mg kg^−1^, Pb = 0.20 mg kg^−1^ and Cr = Not Detected) were applied to 10 kg soil at the rate of 0.5, 1, 2, and 4%, including untreated soil.

### Pot experiment

A pot experiment in the screen house (X = 34.02306 Y = 71.47389), Soil and Environmental Sciences Department, The University of Agriculture Peshawar, was conducted to evaluate biochar and compost's comparative effect on Pb, Cd, and Cr immobilization. Also, maize growth performance to biochar and compost amendments was assessed. The bulk soil used in the experiment was air-dried. A 2 mm diameter stainless steel screen was used to sieve dried soil. The experimental soil was spiked with Pb, Cd, and Cr heavy metals with each concentration of 20, 10, and 20 mg kg^−1^, respectively, and regularly mixed for one week to even dispersion heavy metals. During the application of heavy metals in soil, the concentration of heavy metals in biochar, compost and soil were also kept in mind. All pots irrigated with freshwater were kept for four days, and then maize seeds were planted in each pot. Per pot, 10 seeds were planted. All pots were treated at recommended N and P fertilizers (120 and 90 kg ha^−1^). Nitrogen dose was applied in split, 50% at the sowing time and remaining 50% after 4 weeks. The experiment was conducted in a completely randomized design, replicated three times. After successful germination, thinning was done, and 5 plants were retained for experimental evaluation. Maize plants were irrigated during a growing period to maintain 60% FC as described by^58^.

### Soil analysis

Soil samples were taken from each pot when all plants were harvested. The soil samples were picked in begs and brought to the laboratory for analysis. Soil pH and EC were determined by using pH and EC meter in 1:5 soil water extract. In detail, for the determination of pH and EC in soil, a suspension was prepared by taking 10 g of soil sample and adding 50 ml of distilled water in a conical flask. The mixture was kept on a shaker for 30 min. After shaking the suspension was filtered through Whatman-42 filter paper. The pH and EC were determined through pH and EC meter.

AB-DTPA extractable heavy metals concentrations were measured by the methods described by Soltanpour^57^. A 20 mL AB-DTPA solution was added into 10 g soil in a centrifuge tube and suspension was shaken for 2 h. The suspension was filtered through Whatman No. 42 filter paper and kept at room temperature. Heavy metals extract was determined through spectrophotometer (SHIMADZU AA-6300). Soil total nitrogen was analyzed by Kjeldahl apparatus techniques as described by Bremner et al.^54^. Soil organic matter was determined by the method given by Nelson and Sommers^53^. Soil texture was determined by the hydrometer method^50^.

### Plant analysis

After 60 days of sowing, plants were measured for height and cut to weight shoot and root biomass. Distilled water was used for washing of shoot and root. Till constant weight, shoot and root were kept in an oven at 70 °C, and dry weight was recorded. Photosynthetic pigments and gas exchange parameters were measured according to the procedure as recently reported by Rehman et al.^59^.

### Heavy metals determination in plants

To analyze heavy metals in plants, samples were washed with deionized water. Drying was done in the oven at 70 °C for 48 h. The 0.5 g of dry plant’s samples were taken with 10 mL of nitric acid. After overnight, 4 mL of perchloric acid was mixed. Then digestion was done on the electric hot plate until the brown colour become vanished. The atomic absorption spectrophotometer (SHIMADZU AA-6300) was used to assess heavy metals in each sample.

### Statistical analysis

The recorded data of the experiment was expressed as the mean data. Analysis of variance was prepared using Statistix 8.1. The significant difference between treatments was differentiated by the statistical test known as Least Significant Difference (LSD) with p-value less than 0.05^60^.

### Plant material collection and use permission

No permission is required for plant material as it was purchased from certified dealer of local area.

### Ethics approval and consent to participate

We all declare that manuscripts reporting studies do not involve any human participants, human data, or human tissue. So, it is not applicable.

### Complies with international, national and/or institutional guidelines

Experimental research and field studies on plants (either cultivated or wild), comply with relevant institutional, national, and international guidelines and legislation.
